# Optimal Channel Training Design for Secure Short-Packet Communications

**DOI:** 10.3390/s23031068

**Published:** 2023-01-17

**Authors:** Dechuan Chen, Jin Li, Jianwei Hu, Xingang Zhang, Shuai Zhang

**Affiliations:** 1 College of Physics and Electronic Engineering, Nanyang Normal University, Nanyang 473061, China; 2 Key Lab of Broadband Wireless Communication and Sensor Network Technology (Nanjing University of Posts and Telecommunications), Ministry of Education, Nanjing 210003, China; 3 National Key Laboratory for Complex Systems Simulation, Beijing 100101, China; 4 College of Computer Science and Technology, Nanyang Normal University, Nanyang 473061, China

**Keywords:** physical layer security, short-packet communications, channel training, average secrecy throughput

## Abstract

Physical layer security is a promising technique to ensure the confidentiality of short-packet communications, since no additional channel uses are needed. Motivated by the fact of finite coding blocklength in short-packet communications, we attempt to investigate the problem of how many the channel uses utilized for channel training should be allocated to perform secure communications. Based on the finite blocklength information theory, we derive a closed-form expression to approximate the average achievable secrecy throughput. To gain more insights, we also present the asymptotic average secrecy throughput under two special cases, i.e., high signal-to-noise ratio (SNR) and infinite blocklength. Moreover, we determine the optimal channel training length to maximize the average secrecy throughput under the reliability constraint and given blocklength. Numerical results are provided to validate the analysis and demonstrate that the performance gain achieved by the optimal channel training length is remarkable, relative to other benchmark schemes.

## 1. Introduction

Short-packet communications are recognized as a prominent technique for the fifth generation (5G) and next generation communication networks since they can fulfill two stringent quality-of-service (QoS) requirements, i.e., ultra-low latency and super-high reliability [1,2,3]. The typical size of packet in short-packet communications is only hundreds of bits (e.g., 80–160 bits of industrial manufacturing and control systems [4]). Due to the limited packet length, the decoding error probability in short-packet communications cannot be reduced to arbitrarily low. Motivated by this, block-error-rate as a proper performance metric was developed in [5] to measure the performance of short-packet communication systems, and the block-error-rate of the system increases with the decrease of the blocklength. Since then, short-packet communications have attracted considerable attention from both academia and the industry.

Due to the unchangeable open nature of the wireless transmission medium, security is also a challenging issue for short-packet communications [6,7]. Conventionally, the security is enhanced by encryption techniques, which are deployed at the network layer of communication systems. However, all cryptographic measures require more overhead for encryption and decryption and increase latency imposed, which may not be applicable for short-packet communications [8]. As an alternative to cryptography, physical layer security technique is more appealing for short-packet communications since no additional channel uses are needed [9].

Physical layer security has been well investigated in the existing literature, where the coding blocklength is sufficiently large for achieving the secrecy capacity [10,11]. For short-packet communications, ref. [12] derived the maximal secret communication rate subject to reliability and security constraints. Subsequently, ref. [8] studied the secrecy throughput performance with an external multi-antenna eavesdropper, and found the optimal blocklength to maximize the secrecy throughput under reliability and latency constraints. Moreover, considering both reliability and security, ref. [13] established the outage probability and effective throughput to analyze the performance of secure short-packet communications. The authors in [9] considered a multiuser downlink network in the presence of an eavesdropper and developed efficient methods to solve the total transmit power minimization and weighted throughput maximization problems. Packet replication and interface diversity schemes were employed in [14] to improve the secure spectral efficiency, where eavesdroppers are randomly distributed according to Poisson point processes. In [15], the spectrum sensing blocklength and transmission blocklength were jointly optimized to maximize the secrecy throughput. In order to achieve both high spectral efficiency and low communication delay, incorporating short-packet communications with non-orthogonal multiple access (NOMA) networks was investigated in [16,17,18].

It is worth noting that the previous studies on physical layer security with finite blocklength assumed perfect channel state information (CSI) for communications. However, in most realistic scenarios, perfect CSI may not be easy to obtain due to the feedback delay, channel estimation errors, and limited feedback rate. Against this background, ref. [19] addressed the secrecy throughput of full-duplex multiuser multiple-input-multiple-output (MIMO) networks with short packets, where the impacts of imperfect CSI, co-channel interference and self interference are jointly considered. In [20], the optimal power control policy maximizing achievable secrecy rate under the queueing delay requirement was carried out with channel estimation error. It is noted that these studies in [19,20] have not considered explicit channel training schemes for channel estimation. In fact, the channel estimation error is closely related to channel training schemes, e.g., pilot length and pilot power. In particular, the pilot length significantly affects the overall performance of short-packet transmission systems [2]. In [21], the authors presented a pilot-assisted secure short-packet communications with randomly distributed eavesdroppers and characterized the reliability and security performance by transmission error probability and intercept probability, respectively. Furthermore, ref. [22] optimized the pilot length by an iterative algorithm to maximize the achievable effective secrecy rate of the system. It is further noted that the optimal pilot length that maximizes the secrecy rate has no closed-form solution in [22]. Although their results provide useful insights, the computational complexity of the iterative search algorithm is relatively high. On the other hand, the impact of the pilot length on the secrecy throughput of short-packet communications has not been examined thus far.

Motivated by the above considerations, we investigate the channel training design for secure short-packet communications, where a source transmits pilot symbols before its information transmission to enable channel estimation at a destination. In order to maximize the secrecy throughput of the considered system, the number of channel uses allocated to channel training and data transmission need to be carefully optimized. The main contributions of this paper are summarized as follows:

- Based on the finite blocklength information theory, we derive a closed-form expression to approximate the average achievable secrecy throughput, which provides an efficient means to comprehensively evaluate the impact of key system parameters, e.g., the channel training length and blocklength, on the latency-reliability tradeoff.
- To achieve additional insights on the application of the channel training scheme for secure short-packet communications into the practical design, we also present the asymptotic closed-form expressions for the average secrecy throughput under two special cases, i.e., high signal-to-noise ratio (SNR) and infinite blocklength.
- We determine the optimal channel training length to maximize the average secrecy throughput under the reliability constraint and given blocklength. Numerical results demonstrate the performance gain achieved by the optimal channel training length is remarkable relative to the fixed-ratio channel training length and fixed channel training length schemes.

The remainder of this paper is organized as follows. In Section 2, we describe the secure short-packet communication system based on the channel training scheme. In Section 3, we present the closed-form expression to approximate the average achievable secrecy throughput, provide the high SNR and infinite blocklength analyses for the average secrecy throughput, and determine the optimal channel training length to maximize the average secrecy throughput. Finally, we respectively give numerical results and conclusions in Section 4 and Section 5.

## 2. System Model

**Figure 1:**
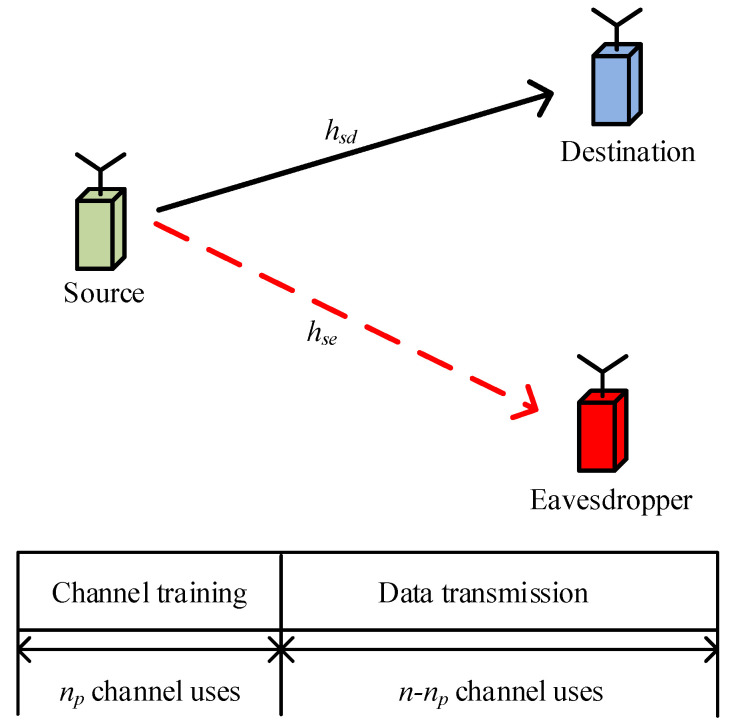
System model and packet structure for short-packet communications.

In this paper, we consider a secure short-packet communication system as shown in Figure 1, in which a source sends confidential short packets to a destination in the presence of a passive eavesdropper. Due to size limitation, each node is equipped with a single antenna. The channels from the source to the destination and the eavesdropper are subject to independent quasi-static Rayleigh fading, which means that the channel coefficients remain static during a coherence slot (*n* channel uses) and vary independently from one coherence slot to the next [22,23,24].

In each short-packet transmission, the source conveys *L* information bits over *n* channel uses to the destination. In order to support the high reliability requirement, we consider a two-phase training-based transmission scheme, which contains a channel training phase and a data transmission phase. In the channel training phase, the source transmits a predefined pilot sequence of $n_{p}$ channel uses to enable channel estimation at the receiver. In the data transmission phase, the source utilizes the remaining $n-n_{p}$ channel uses for information transmission. Thus, the received signal vectors at the destination and the eavesdropper are, respectively, given by

(1) $$y_{d}=h_{sd}x+n_{d},$$

(2) $$y_{e}=h_{se}x+n_{e},$$

where $h_{sd}∼CN\left(0,{\bar{\gamma }}_{sd}\right)$ is the channel coefficient between the source and the destination, $h_{se}∼CN\left(0,{\bar{\gamma }}_{se}\right)$ is the channel coefficient between the source and the eavesdropper, x is the transmitted signal vector from the source, $n_{d}$ and $n_{e}$ are the additive white Gaussian noise (AWGN) with zero-mean and variance $N_{0}$ at the destination and the eavesdropper, respectively. After receiving the signals, both the destination and the eavesdropper estimate their channels by the minimum mean-square error (MMSE) estimator and then decode the data. As such, the actual channel coefficient can be denoted as the sum of the estimated channel and the estimation error. According to [25], we have

(3) $$h_{sd}={\hat{h}}_{sd}+{\tilde{h}}_{sd},$$

(4) $$h_{se}={\hat{h}}_{se}+{\tilde{h}}_{se},$$

where ${\hat{h}}_{sd}∼CN\left(0,\frac{\rho_{p}n_{p}{\bar{\gamma }}_{sd}^{2}}{\rho_{p}n_{p}{\bar{\gamma }}_{sd}+N_{0}}\right)$ is the estimated value of $h_{sd}$, ${\tilde{h}}_{sd}∼CN\left(0,\frac{{\bar{\gamma }}_{sd}N_{0}}{\rho_{p}n_{p}{\bar{\gamma }}_{sd}+N_{0}}\right)$ is the estimation error of $h_{sd}$, ${\hat{h}}_{se}∼CN\left(0,\frac{\rho_{p}n_{p}{\bar{\gamma }}_{se}^{2}}{\rho_{p}n_{p}{\bar{\gamma }}_{se}+N_{0}}\right)$ is the estimated value of $h_{se}$, ${\tilde{h}}_{se}∼CN\left(0,\frac{{\bar{\gamma }}_{se}N_{0}}{\rho_{p}n_{p}{\bar{\gamma }}_{se}+N_{0}}\right)$ is the estimation error of $h_{se}$, and $\rho_{p}$ is the average power of the pilot symbols.

The destination and the eavesdropper use the estimated channel for information reception. Thus, in the data transmission phase, the received signal vectors at the destination and the eavesdropper are, respectively, rewritten as

(5) $$y_{d}=\sqrt{\rho_{d}}{\hat{h}}_{sd}x_{d}+\sqrt{\rho_{d}}{\tilde{h}}_{sd}x_{d}+n_{d},$$

(6) $$y_{e}=\sqrt{\rho_{d}}{\hat{h}}_{se}x_{d}+\sqrt{\rho_{d}}{\tilde{h}}_{se}x_{d}+n_{e},$$

where $\rho_{d}$ is the average power of the data symbols, and $x_{d}$ is the data symbols. The actual instantaneous SNRs for information reception at the destination and the eavesdropper can be, respectively, given by

(7) $$\gamma_{sd}=\frac{\rho_{d}{\left|{\hat{h}}_{sd}\right|}^{2}}{\rho_{d}{\left|{\tilde{h}}_{sd}\right|}^{2}+N_{0}},$$

(8) $$\gamma_{se}=\frac{\rho_{d}{\left|{\hat{h}}_{se}\right|}^{2}}{\rho_{d}{\left|{\tilde{h}}_{se}\right|}^{2}+N_{0}}.$$

We assume that the source uses a fraction α of the total power for data transmission and the remaining 1−α portion for channel training, where α is the power allocation factor. Thus, we have

(9) $$\rho_{d}\left(n-n_{p}\right)=\alpha \rho n,\rho_{p}n_{p}=\left(1-\alpha \right)\rho n,$$

where ρ is the average power of all the transmitted symbols at the source. Then, the instantaneous SNRs at the destination and the eavesdropper can be, respectively, rewritten as

(10) $$\gamma_{sd}=\frac{\alpha \rho n{\left|{\hat{h}}_{sd}\right|}^{2}}{\alpha \rho n{\left|{\tilde{h}}_{sd}\right|}^{2}+N_{0}\left(n-n_{p}\right)},$$

(11) $$\gamma_{se}=\frac{\alpha \rho n{\left|{\hat{h}}_{se}\right|}^{2}}{\alpha \rho n{\left|{\tilde{h}}_{se}\right|}^{2}+N_{0}\left(n-n_{p}\right)}.$$

Since the eavesdropper’s estimation error is typically unknown to the source, it is necessary to design a robust approach for the worst-case scenario. That is, there is no estimation error at the eavesdropper. Then, the actual instantaneous SNR at the eavesdropper can be rewritten as

(12) $$\gamma_{se}=\frac{\alpha \rho n{\left|h_{se}\right|}^{2}}{N_{0}\left(n-n_{p}\right)}.$$

Based on [8], the achievable secrecy rate of the considered system with the blocklength *n* and channel training length $n_{p}$ for a given constraint on the decoding error probability ϵ and a secrecy constraint on the information leakage δ can be approximated as

(13) $$\begin{array}{c} R_{s}\left(n,n_{p},\epsilon ,\delta \right)\\ =\left\{\begin{array}{cc} C_{s}-\sqrt{\frac{V_{sd}}{n-n_{p}}}\frac{Q^{-1}\left(\epsilon \right)}{\ln2}-\sqrt{\frac{V_{se}}{n-n_{p}}}\frac{Q^{-1}\left(\delta \right)}{\ln2}, & \gamma_{sd}>\gamma_{se},\\ 0, & \gamma_{sd}\leq \gamma_{se}, \end{array}\right. \end{array}$$

where $C_{s}=\log_{2}\left(1+\gamma_{sd}\right)-\log_{2}\left(1+\gamma_{se}\right)$ is the secrecy capacity with infinite blocklength, $V_{x}=1-{\left(1+\gamma_{x}\right)}^{-2}$, $x\in \left\{sd,se\right\}$, is the channel dispersion, and $Q^{-1}\left(\cdot \right)$ is the inverse *Q*-function $Q\left(x\right)=\int_{x}^{\infty }\frac{1}{\sqrt{2\pi }}e^{-\frac{t^{2}}{2}}dt$.

## 3. Secrecy Performance Analysis

In this section, we investigate the average secrecy throughput performance of the short-packet communication system with the channel training scheme. Then, we focus on the asymptotic analysis for the average secrecy throughput. Finally, we determine the optimal channel training length to maximize the average secrecy throughput under the reliability constraint and given blocklength.

### 3.1. Secrecy Throughput Approximation

The secrecy throughput in short-packet communications is defined as the average secrecy rate where the data packet is reliably transmitted subject to a certain secrecy constraint. Mathematically, the average secrecy throughput of the considered system is formulated as

(14) $$T=E{}_{\gamma_{sd},\gamma_{se}}\left(\frac{L}{n-n_{p}}\left(1-\epsilon \right)\right)=\frac{L}{n-n_{p}}\left(1-\bar{\epsilon }\right)$$

where $\bar{\epsilon }=E{}_{\gamma_{sd},\gamma_{se}}\left(\epsilon \right)$ is the average decoding error probability. When $\gamma_{sd}>\gamma_{se}$, the decoding error probability at the destination can be characterized by $\epsilon =Q\left(\sqrt{\frac{n-n_{p}}{V_{sd}}}\left(\ln\frac{1+\gamma_{sd}}{1+\gamma_{se}}-\sqrt{\frac{V_{se}}{n-n_{p}}}Q^{-1}\left(\delta \right)-\frac{L}{n-n_{p}}\ln2\right)\right)$. When $\gamma_{sd}\leq \gamma_{se}$, the achievable secrecy rate is zero and we set ϵ=1. The average secrecy throughput in (14) can be further derived as

(15) $$T=\frac{L}{n-n_{p}}\int_{0}^{\infty }\Psi \left(y\right)f_{\gamma_{se}}\left(y\right)dy,$$

where $\Psi \left(y\right)=\int_{y}^{\infty }\left(1-\epsilon_{\gamma_{sd}\left|\gamma_{se}=y\right.}\left(x\right)\right)f_{\gamma_{sd}}\left(x\right)dx$, $\epsilon_{\gamma_{sd}\left|\gamma_{se}=y\right.}\left(\cdot \right)$ is the conditional decoding error probability conditioned on $\gamma_{se}=y$, and $f_{\gamma_{se}}\left(y\right)$ is the probability density function (PDF) of $\gamma_{se}$. In order to calculate the double integral in (15), we propose to use the first-order approximation of $\epsilon_{\gamma_{sd}\left|\gamma_{se}=y\right.}\left(x\right)$ as follows

(16) $$\begin{array}{cc} \; & \epsilon_{\gamma_{sd}\left|\gamma_{se}=y\right.}\left(x\right)\approx P_{\gamma_{sd}\left|\gamma_{se}\right.}\left(x\right)\\ \; & =\left\{\begin{array}{cc} 1, & x<\frac{1}{2k}+x_{0},\\ \frac{1}{2}+k\left(x-x_{0}\right), & x\in \left[\frac{1}{2k}+x_{0},-\frac{1}{2k}+x_{0}\right],\\ 0, & x>-\frac{1}{2k}+x_{0}, \end{array}\right. \end{array}$$

where $x_{0}=e^{\sqrt{\frac{V_{se}}{n-n_{p}}}Q^{-1}\left(\delta \right)+\frac{L}{n-n_{p}}\ln2}\left(1+\gamma_{se}\right)-1$ and $k={\left.\frac{d\epsilon_{\gamma_{sd}\left|\gamma_{se}=y\right.}\left(x\right)}{dx}\right|}_{x=x_{0}}=-\sqrt{\frac{n-n_{p}}{2\pi x_{0}\left(x_{0}+2\right)}}$.

Based on the fact that $\epsilon_{\gamma_{sd}\left|\gamma_{se}=y\right.}\left(x\right)>1/2$ when x<y, the integral $\Psi \left(y\right)$ can be further simplified by changing the lower limit from *y* to 0. Therefore, we have

(17) $$\begin{array}{cc} \Psi \left(y\right) & \approx \int_{0}^{\infty }\left(1-\epsilon_{\gamma_{sd}\left|\gamma_{se}=y\right.}\left(x\right)\right)f_{\gamma_{sd}}\left(x\right)dx\\ \; & \approx 1-\int_{0}^{\infty }P_{\gamma_{sd}\left|\gamma_{se}\right.}\left(x\right)f_{\gamma_{sd}}\left(x\right)dx\\ \; & =1+k\int_{\frac{1}{2k}+x_{0}}^{-\frac{1}{2k}+x_{0}}F_{\gamma_{sd}}\left(x\right)dx, \end{array}$$

where $F_{\gamma_{sd}}\left(x\right)$ and $f_{\gamma_{sd}}\left(x\right)$ are respectively the cumulative distribution function (CDF) and PDF of $\gamma_{sd}$. It is important to point out that $\left|k\right|$ is an increasing function of *n*. When *n* is in the moderate blocklength region, i.e., $10^{2}\leq n\leq 10^{3}$, which is really important to short-packet communications, the integral interval $\left[\frac{1}{2k}+x_{0},-\frac{1}{2k}+x_{0}\right]$ is generically small. Therefore, with the help of the first order Riemann integral approximation, we further approximate (17) as

(18) $$\Psi \left(y\right)\approx 1-F_{\gamma_{sd}}\left(x_{0}\right).$$

According to (10) and (12), the CDF of $\gamma_{sd}$ and the PDF of $\gamma_{se}$ can be, respectively, formulated as

(19) $$F_{\gamma_{sd}}\left(x\right)=1-\frac{\left(1-\alpha \right)\rho n{\bar{\gamma }}_{sd}^{2}e^{-\frac{xN_{0}\left(n-n_{p}\right)\left(\left(1-\alpha \right)\rho n{\bar{\gamma }}_{sd}+N_{0}\right)}{\alpha \left(1-\alpha \right)\rho^{2}n^{2}{\bar{\gamma }}_{sd}^{2}}}}{x{\bar{\gamma }}_{sd}N_{0}+\left(1-\alpha \right)\rho n{\bar{\gamma }}_{sd}^{2}},$$

and

(20) $$f_{\gamma_{se}}\left(y\right)=\frac{N_{0}\left(n-n_{p}\right)}{\alpha \rho n{\bar{\gamma }}_{se}}e^{-\frac{yN_{0}\left(n-n_{p}\right)}{\alpha \rho n{\bar{\gamma }}_{se}}}.$$

By applying (18)–(20) into (15), we have

(21) $$\begin{array}{cc} T & \approx \frac{\left(1-\alpha \right)LN_{0}{\bar{\gamma }}_{sd}^{2}}{\alpha {\bar{\gamma }}_{se}}\int_{0}^{\infty }\frac{e^{-\left(\frac{x_{0}\left(y\right)N_{0}\left(n-n_{p}\right)}{\alpha \rho n{\hat{\gamma }}_{sd}}+\frac{yN_{0}\left(n-n_{p}\right)}{\alpha \rho n{\bar{\gamma }}_{se}}\right)}}{x_{0}\left(y\right){\bar{\gamma }}_{sd}N_{0}+\left(1-\alpha \right)\rho n{\bar{\gamma }}_{sd}^{2}}dy\\ \; & \approx \frac{\left(1-\alpha \right)LN_{0}{\bar{\gamma }}_{sd}^{2}}{\alpha {\bar{\gamma }}_{se}}\left(\underset{\Xi_{1}}{\underset{︸}{\int_{0}^{M_{1}}\frac{e^{-\left(\frac{x_{0}\left(y\right)N_{0}\left(n-n_{p}\right)}{\alpha \rho n{\hat{\gamma }}_{sd}}+\frac{yN_{0}\left(n-n_{p}\right)}{\alpha \rho n{\bar{\gamma }}_{se}}\right)}}{x_{0}\left(y\right){\bar{\gamma }}_{sd}N_{0}+\left(1-\alpha \right)\rho n{\bar{\gamma }}_{sd}^{2}}dy}}\right.\\ \; & \;\;\;\;\;\left.+\underset{\Xi_{2}}{\underset{︸}{\int_{M_{1}}^{\infty }\frac{e^{-\left(\frac{\left(\varpi_{1}y+\varpi_{1}-1\right)N_{0}\left(n-n_{p}\right)}{\alpha \rho n{\hat{\gamma }}_{sd}}+\frac{yN_{0}\left(n-n_{p}\right)}{\alpha \rho n{\bar{\gamma }}_{se}}\right)}}{\left(\varpi_{1}y+\varpi_{1}-1\right){\bar{\gamma }}_{sd}N_{0}+\left(1-\alpha \right)\rho n{\bar{\gamma }}_{sd}^{2}}dy}}\right), \end{array}$$

where ${\hat{\gamma }}_{sd}=\frac{\left(1-\alpha \right)\rho n{\bar{\gamma }}_{sd}^{2}}{\left(1-\alpha \right)\rho n{\bar{\gamma }}_{sd}+N_{0}}$, $\varpi_{1}=e^{\frac{Q^{-1}\left(\delta \right)}{\sqrt{n-n_{p}}}+\frac{L}{n-n_{p}}\ln2}$ and $M_{1}$ is a sufficiently large parameter to ensure $V_{se}\approx 1$ when $\gamma_{se}>M_{1}$.

By leveraging Gaussian-Chebyshev quadrature, the integral $\Xi_{1}$ can be approximated as

(22) $$\Xi_{1}\approx \frac{M_{1}}{2}\sum_{m=1}^{M_{2}}\left(\frac{\pi }{M_{2}}f\left(\frac{M_{1}}{2}\left(t_{m}+1\right)\right)\sqrt{1-t_{m}^{2}}\right),$$

where $M_{2}$ is a parameter for the complexity accuracy tradeoff, $f\left(z\right)=\frac{e^{-\left(\frac{x_{0}\left(z\right)N_{0}\left(n-n_{p}\right)}{\alpha \rho n{\hat{\gamma }}_{sd}}+\frac{zN_{0}\left(n-n_{p}\right)}{\alpha \rho n{\bar{\gamma }}_{se}}\right)}}{x_{0}\left(z\right){\bar{\gamma }}_{sd}N_{0}+\left(1-\alpha \right)\rho n{\bar{\gamma }}_{sd}^{2}}$ with ${\left.x_{0}\left(z\right)=x_{0}\right|}_{\gamma_{se}=z}$, and $t_{m}=\cos\left(\frac{2m-1}{2M_{2}}\pi \right)$.

According to [26] (3.352.2), the integral $\Xi_{2}$ can be derived as

(23) $$\Xi_{2}=-\frac{e^{\varpi_{2}\varpi_{3}-\frac{\left(\varpi_{1}-1\right)N_{0}\left(n-n_{p}\right)}{\alpha \rho n{\hat{\gamma }}_{sd}}}}{\varpi_{1}{\bar{\gamma }}_{sd}N_{0}}Ei\left(-M_{1}\varpi_{2}-\varpi_{2}\varpi_{3}\right),$$

where $Ei\left(\cdot \right)$ is the exponential integral function, $\varpi_{2}=\frac{\varpi_{1}N_{0}\left(n-n_{p}\right)}{\alpha \rho n{\hat{\gamma }}_{sd}}+\frac{N_{0}\left(n-n_{p}\right)}{\alpha \rho n{\bar{\gamma }}_{se}}$ and $\varpi_{3}=\frac{\left(\varpi_{1}-1\right)N_{0}+\left(1-\alpha \right)\rho n{\bar{\gamma }}_{sd}}{\varpi_{1}N_{0}}$.

The average secrecy throughput of the considered system with short-packet communications can be directly obtained by substituting (22) together with (23) into (21).

### 3.2. High SNR Regime

To further characterize the impact of key system parameters on the average secrecy throughput, we focus on the asymptotic average secrecy throughput in the high SNR regime, where the average transmit power ρ at the source approaches infinity. When ρ→∞, we know that the estimation error approaches zero and $E\left(\gamma_{se}\right)$ approaches infinity. According to (21), the average secrecy throughput in the high SNR regime of the considered system with finite blocklength can be simplified as

(24) $$\begin{array}{c} T^{\rho \to \infty }=\frac{L}{n-n_{p}}\left(1-{\bar{\epsilon }}^{\rho \to \infty }\right), \end{array}$$

where ${\bar{\epsilon }}^{\rho \to \infty }=1-\frac{\left(\alpha -1\right)\left(n-n_{p}\right){\bar{\gamma }}_{sd}}{\alpha \varpi_{1}{\bar{\gamma }}_{se}}e^{\frac{\left(1-\alpha \right)\left(n-n_{p}\right)}{\alpha }\left(1+\frac{{\bar{\gamma }}_{sd}}{\varpi_{1}{\bar{\gamma }}_{se}}\right)}$$Ei\left(\frac{\left(\alpha -1\right)\left(n-n_{p}\right)}{\alpha }\left(1+\frac{{\bar{\gamma }}_{sd}}{\varpi_{1}{\bar{\gamma }}_{se}}\right)\right)$. An important observation from (24) is that the average decoding error probability cannot reduce to zero even when the transmit power at the source approaches infinity. This is because not only the destination but also the eavesdropper will benefit from increasing the transmit power. Moreover, we know that the average secrecy throughput in the high SNR regime is dependent on the power allocation factor between channel training and data transmission.

### 3.3. The Class Case with Infinite Blocklength

To further understand the connection between finite blocklength and infinite blocklength, we turn our attention to the classical case with infinite blocklength. When N→∞, we know that ϵ→0 as long as $\gamma_{sd}>\gamma_{se}$ (otherwise ϵ→1). Thus, the average secrecy throughput of the considered system with infinite blocklength can be expressed as

(25) $$\begin{array}{cc} T^{n\to \infty } & =\frac{L}{n-n_{p}}\mathrm{Pr}\left(\gamma_{sd}>\gamma_{se}\right),\\ \; & =\frac{L{\bar{\gamma }}_{sd}\left(\alpha -1\right)}{\alpha {\bar{\gamma }}_{se}}e^{\left(\frac{1}{{\bar{\gamma }}_{sd}}+\frac{1}{{\bar{\gamma }}_{se}}+\frac{N_{0}}{\left(1-\alpha \right)\rho n{\bar{\gamma }}_{sd}^{2}}\right)}\\ \; & \;\;\;\;\;\times e^{\frac{\left(1-\alpha \right)\left(n-n_{p}\right){\bar{\gamma }}_{sd}}{\alpha }}Ei\left(\frac{\left(\alpha -1\right)\left(n-n_{p}\right){\bar{\gamma }}_{sd}}{\alpha }\right.\\ \; & \;\;\;\;\;\left.\left(\frac{1}{{\bar{\gamma }}_{sd}}+\frac{1}{{\bar{\gamma }}_{se}}+\frac{N_{0}}{\left(1-\alpha \right)\rho n{\bar{\gamma }}_{sd}^{2}}\right)\right). \end{array}$$

From (25), it is worth noting that $T^{n\to \infty }\to 0$ due to the fact that $e^{x}Ei\left(-x\right)\to 0$ as x→∞. This is because the transmission rate $\frac{L}{n-n_{p}}\to 0$ as n→∞. However, when $\gamma_{sd}>\gamma_{se}$, the secrecy capacity of the considered system is not zero.

### 3.4. Optimal Transmission Design

To maximize the average secrecy throughput, the designers have to choose the suitable channel training length in a coherence slot. This is due to the fact that the channel estimation becomes more accurate and the destination can decode more information bits reliably as the channel training length increases. However, this will reduce the duration for data transmission at the same time, which leads to the degradation of the average secrecy throughput. The optimization of $n_{p}$ maximizing the average secrecy throughput under the reliability constraint and given blocklength can be formulated as

(26a) $$\begin{array}{cc} \max_{n_{p}}\;\;\;\; & T, \end{array}$$

(26b) $$\begin{array}{cc} s.t.\;\;\;\; & \bar{\epsilon }\leq \epsilon_{\max}, \end{array}$$

(26c) $$\begin{array}{cc} \;\;\;\; & 0\leq n_{p}\leq n, \end{array}$$

(26d) $$\begin{array}{cc} \;\;\;\; & n_{p}\in N^{+}, \end{array}$$

where $N^{+}$ denotes the non-negative integer set and (26b) denotes the system’s reliability constraint.

In the following, we show that $\bar{\epsilon }$ is a convex increasing function of $n_{p}$ and *T* is a quasi-concave function of $n_{p}$. Based on the Leibniz integral rule, the monotonicity of $\bar{\epsilon }$ with respect to $n_{p}$ is consistent with that of ϵ with respect to $n_{p}$. Taking the first and second derivative of ϵ on $n_{p}$, we have

(27) $$\frac{\partial \epsilon }{\partial n_{p}}=\frac{\partial \epsilon }{\partial \phi }\frac{\partial \phi }{\partial n_{p}},$$

and

(28) $$\frac{\partial^{2}\epsilon }{\partial n_{p}^{2}}=\frac{\partial^{2}\epsilon }{\partial \phi^{2}}{\left(\frac{\partial \phi }{\partial n_{p}}\right)}^{2}+\frac{\partial \epsilon }{\partial \phi }\frac{\partial^{2}\phi }{\partial n_{p}^{2}}$$

where $\phi =\sqrt{\frac{n-n_{p}}{V_{sd}}}\left(\ln\frac{1+\gamma_{sd}}{1+\gamma_{se}}-\sqrt{\frac{V_{se}}{n-n_{p}}}Q^{-1}\left(\delta \right)-\frac{L}{n-n_{p}}\ln2\right)$. For short-packet communications, ϵ is generally much smaller than 0.5. Hence, we have $\phi =Q^{-1}\left(\epsilon \right)>0$, $\frac{\partial \epsilon }{\partial \phi }=-\frac{1}{\sqrt{2\pi }}e^{-\frac{\phi^{2}}{2}}<0$ and $\frac{\partial^{2}\epsilon }{\partial \phi^{2}}=\frac{\phi }{\sqrt{2\pi }}e^{-\frac{\phi^{2}}{2}}>0$. Then, we need to check the sign of $\frac{\partial \phi }{\partial n_{p}}$ and $\frac{\partial^{2}\phi }{\partial n_{p}^{2}}$. To facilitate analysis, we approximate $V_{sd}=V_{se}\approx 1$ and $\ln\frac{1+\gamma_{sd}}{1+\gamma_{se}}\approx \ln\frac{\gamma_{sd}}{\gamma_{se}}\approx \ln\frac{{\left|h_{sd}\right|}^{2}}{{\left|h_{se}\right|}^{2}}$, which is very accurate in the high SNR regime [8]. Then, we have ∂ϕ∂np=−n−nplnhsd2hsd2hse2hse2+Lln22n−np3322<0 and ∂2ϕ∂np2=−n−nplnhsd2hsd2hse2hse2+3Lln24n−np5522<0. Therefore, we state that $\bar{\epsilon }$ is a convex increasing function of $n_{p}$ and *T* is a quasi-concave function of $n_{p}$.

When ${\left.\frac{\partial T}{\partial n_{p}}\right|}_{n_{p}=0}\leq 0$, the optimal channel training length for problem (26) is given by

(29) $$n_{p}^{*}=0.$$

When ${\left.\frac{\partial T}{\partial n_{p}}\right|}_{n_{p}=0}>0$, the optimal channel training length for problem (26) is given by

(30) $$n_{p}^{*}=\left\{\begin{array}{cc} \arg\;\max_{n_{p}\in \left\{\left\lceil n_{p}^{\#}\right\rceil ,\left\lfloor n_{p}^{\#}\right\rfloor \right\}}T, & n_{p}^{\#}<\min\left(\left\lceil n_{p}^{o}\right\rceil ,n\right),\\ \min\left(\left\lceil n_{p}^{o}\right\rceil ,n\right), & n_{p}^{\#}\geq \min\left(\left\lceil n_{p}^{o}\right\rceil ,n\right). \end{array}\right.$$

where $n_{p}^{\#}$ is the solution of $\frac{\partial T}{\partial n_{p}}=0$, $n_{p}^{o}$ is the solution of $\bar{\epsilon }=\epsilon_{\max}$, and $\left\lceil \cdot \right\rceil$ and $\left\lfloor \cdot \right\rfloor$ are the ceiling and floor operations, respectively.

**Proof.**  We first relax the integer constraint in problem (26). Then, the optimal channel training length can be derived directly from the fact that $\bar{\epsilon }$ is a convex increasing function of $n_{p}$ and *T* is a quasi-concave function of $n_{p}$. ☐

## 4. Numerical Results

In this section, we provide simulation and numerical results to demonstrate how the key system parameters, i.e., channel training length and blocklength, impact the average secrecy throughput of the considered system. Unless otherwise stated, the system parameter settings are as follows: L=200, $\delta =10^{-2}$, $N_{0}=1$, $M_{1}=10$ and $M_{2}=20$.

**Figure 2:**
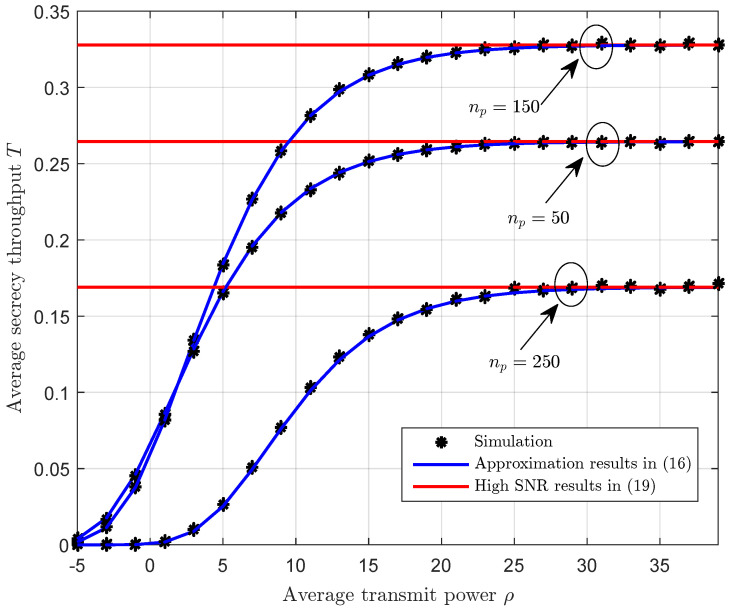
The average secrecy throughput *T* versus the average transmit power ρ with different channel training length $n_{p}$, where α=0.5, n=300, ${\bar{\gamma }}_{sd}=0$ dB, and ${\bar{\gamma }}_{se}=0$ dB.

Figure 2 shows the average secrecy throughput versus the average transmit power with different channel training length. We first observe that the approximation results in (21) coincide well with the Monte-Carlo simulation points, which corroborates the accuracy of the analytical expressions. Second, we observe that the average secrecy throughput increases as the average transmit power increases, and then converges to a constant when the average transmit power is sufficiently large. This is due to the fact that the average secrecy throughput is independent of the average transmit power in the high SNR regime according to (24). Moreover, we observe that the channel training length is not better when the blocklength is fixed.

**Figure 3:**
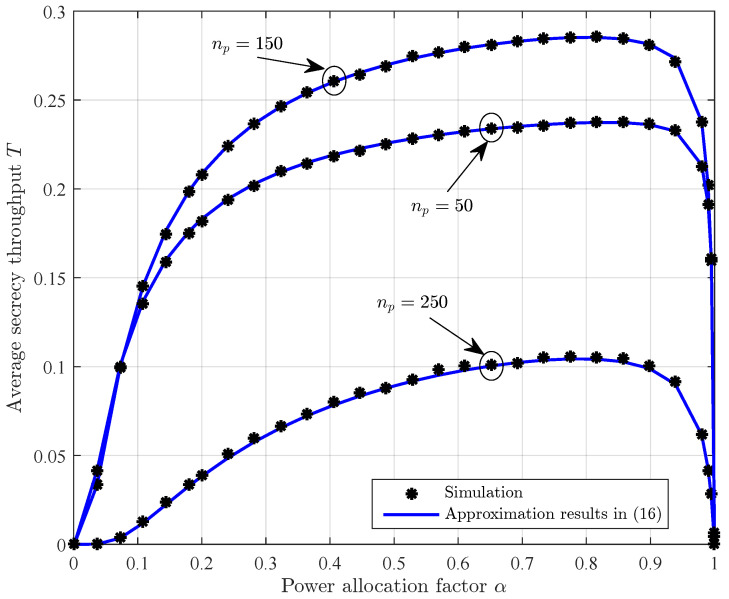
The average secrecy throughput *T* versus the power allocation factor α with different channel training length $n_{p}$, where n=300, ρ=10 dB, ${\bar{\gamma }}_{sd}=0$ dB, and ${\bar{\gamma }}_{se}=0$ dB.

Figure 3 depicts the average secrecy throughput versus the power allocation factor with different channel training length. We observe that the average secrecy throughput increases as the power allocation factor increases from 0 to an optimal value but later, it starts decreasing as the power allocation factor increases from its optimal value. This can be explained as follows. When the power allocation factor is too small, there is less power available for data transmission, which, of course, will result in poor average secrecy throughput. When the power allocation factor is too large, there is less power available for channel training which, consequently, also leads to poor average secrecy throughput. Although an explicit solution for the optimal power allocation factor is intractable due to the complexity of the average secrecy throughput expression, the solution can be obtained offline by numerical search methods, for example, the gradient-based search method.

**Figure 4:**
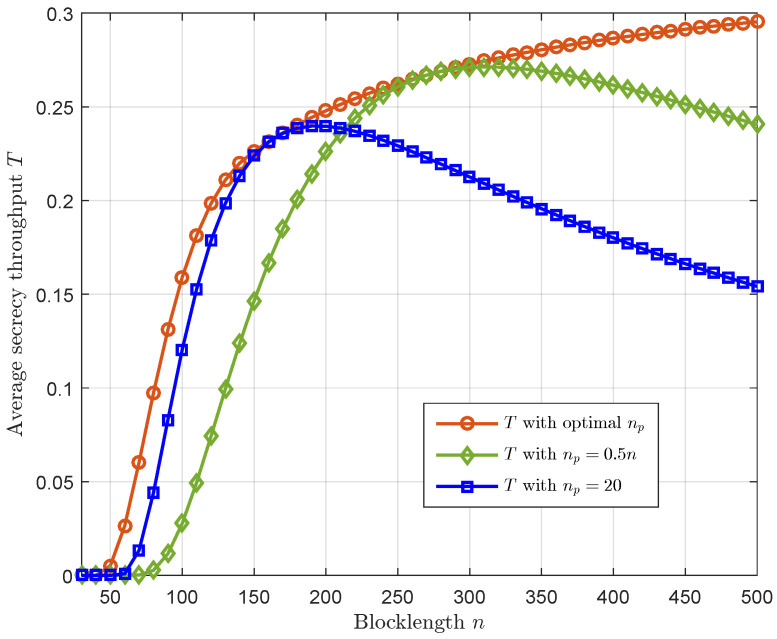
The average secrecy throughput *T* versus the blocklength *n* with α=0.5, ρ=10 dB, ${\bar{\gamma }}_{sd}=0$ dB, and ${\bar{\gamma }}_{se}=0$ dB, where the optimal channel training length is obtained without reliability constraint.

Figure 4 demonstrates that the optimal channel training length can significantly improve the average secrecy throughput of the considered system. To obtain comparable results, we provide the following channel training transmission schemes: (1) Fixed-ratio channel training length, in which the channel training length $n_{p}=0.5n$ is fixed; (2) Fixed channel training length, in which the channel training length $n_{p}=20$ is fixed. It is clear that the average secrecy throughput with the optimal channel training is superior to the two benchmark schemes mentioned above, which implies that the average secrecy throughput of the considered system can be significantly improved via optimizing the channel training length.

**Figure 5:**
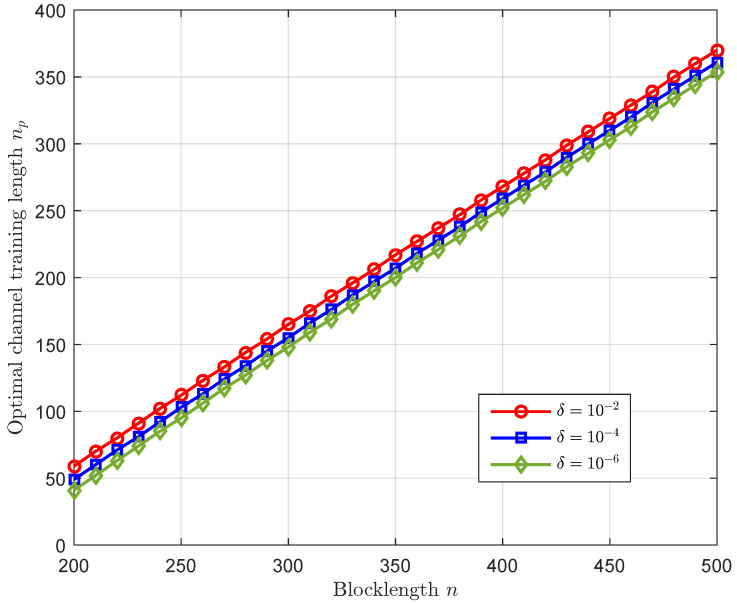
The optimal channel training length $n_{p}$ versus the blocklength *n* with α=0.5, ρ=10 dB, ${\bar{\gamma }}_{sd}=0$ dB, and ${\bar{\gamma }}_{se}=0$ dB, where the optimal channel training length is obtained without reliability constraint.

Figure 5 depicts the optimal channel training length versus the blocklength with different values of δ. We observe that the optimal channel training length that maximizes the average secrecy throughput increases with the increase of the blocklength. Moreover, from Figure 4, one can observe that the average secrecy throughput with the optimal channel training length increases as the blocklength increases. Thus, we can conclude that when the transmission latency constraint is loose, it is favorable to allocate more channel uses for channel estimation to mitigate the decoding error.

**Figure 6:**
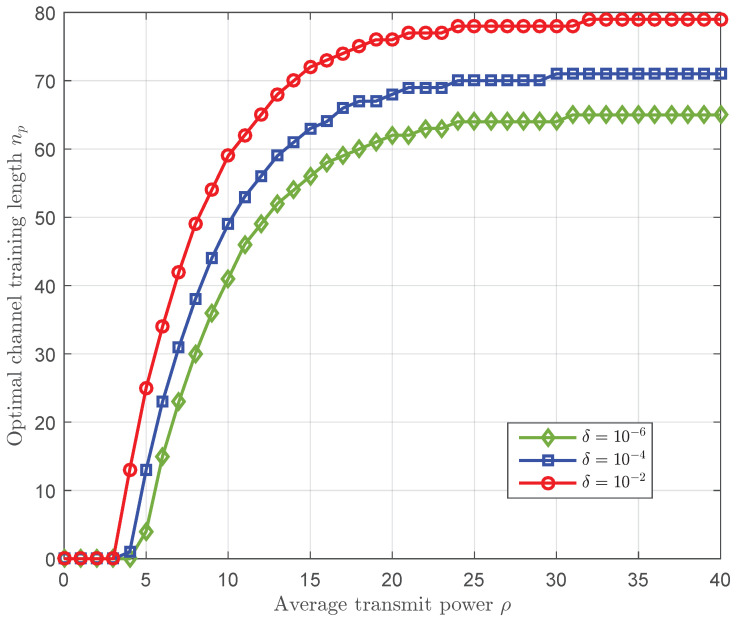
The optimal channel training length $n_{p}$ versus the average transmit power ρ with α=0.5, n=200, ${\bar{\gamma }}_{sd}=0$ dB, and ${\bar{\gamma }}_{se}=0$ dB, where the optimal channel training length is obtained without reliability constraint.

Figure 6 plots the optimal channel training length versus the average transmit power with different values of δ. We first observe that for a fixed δ, the optimal channel training length increases as the average transmit power increases. Moreover, we also observe that for a fixed average transmit power, the optimal channel training length increases as δ increases. This is because when either the average transmit power or the tolerance of the information leakage δ increases, the probability of decoding error decreases and the optimal channel training length becomes larger in order to support higher transmission rate.

**Figure 7:**
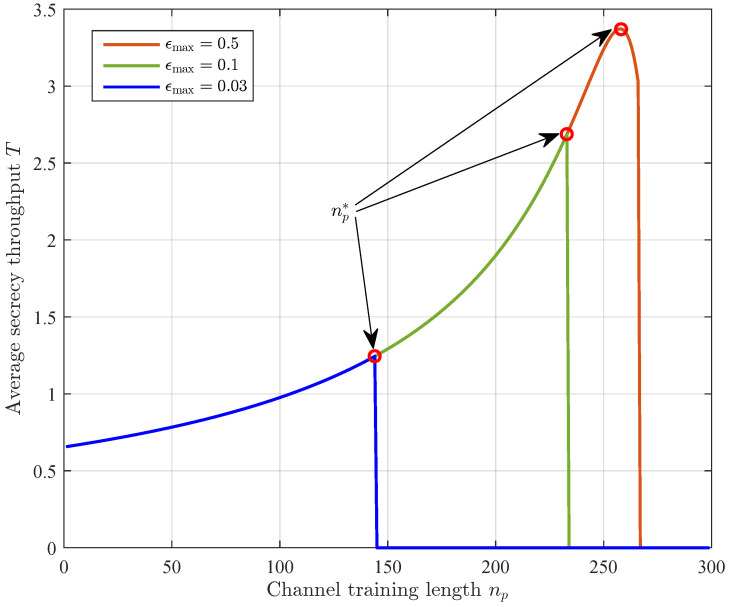
The average secrecy throughput *T* versus the channel training length $n_{p}$ under the reliability constraint, where α=0.5, n=300, ρ=10 dB, ${\bar{\gamma }}_{sd}=20$ dB, and ${\bar{\gamma }}_{se}=0$ dB.

Figure 7 plots the average secrecy throughput versus the channel training length with different values of $\epsilon_{\max}$. We first observe that strengthening the reliability constraint, i.e., reducing $\epsilon_{\max}$, decreases the average secrecy throughput. We further observe that the optimal channel training length maximizing the average secrecy throughput depends on the value of $\epsilon_{\max}$. In particular, when $\epsilon_{\max}$ is small, the average secrecy throughput monotonically increases as the channel training length increases, such that the optimal channel training length is at the right boundary. When $\epsilon_{\max}$ becomes larger, the average secrecy throughput first increases and then decreases as the channel training length increases, and the optimal channel training length is the one from $\left\{\left\lceil \frac{\partial T}{\partial n_{p}}=0\right\rceil ,\left\lfloor \frac{\partial T}{\partial n_{p}}=0\right\rfloor \right\}$ that yields the largest average secrecy throughput.

## 5. Conclusions

In this paper, we investigated the average secrecy throughput of a short-packet communication system with a two-phase training-based transmission scheme. Based on the finite blocklength information theory, the average secrecy throughput has been approximated in closed-form, which quantitatively reveals the impact of channel training length on the the tradeoff between reliability and transmission latency under a secrecy constraint. In addition, we also derived simple asymptotic results for the average secrecy throughput to offer valuable insights into practical design. Finally, the optimal channel training length under the reliability constraint and given blocklength was obtained, and the simulation results demonstrated that the performance gain achieved by the optimal channel training length is remarkable, relative to the fixed-ratio channel training length and fixed channel training length schemes.

## Data Availability

Not applicable.
